# A Child With a Glasgow Coma Scale Score of 4 After Near-Hanging: A Case Report on Treatment Without Intubation and Targeted Temperature Management

**DOI:** 10.7759/cureus.39581

**Published:** 2023-05-27

**Authors:** Koji Kanno, Yuji Yamagami, Tomoya Hanada, Madoka Iwahashi, Yusuke Ito

**Affiliations:** 1 Department of Pediatric Emergency and Critical Care Medicine, Hyogo Prefectural Amagasaki General Medical Center, Amagasaki, JPN

**Keywords:** prehospital emergency medicine, glasgow coma scale score, intubation, targeted temperature management, hanging, near hanging

## Abstract

While prehospital medical interventions are evolving and improving survival rates, the evidence for adequate early prognostic assessment is often insufficient. A 12-year-old Japanese child was found hanging on the roof of his home. After being rescued by his mother, he was transported by an ambulance and a rapid response car (RRC) with doctors, nurses, and paramedics on board, and admitted to our hospital. His initial Glasgow Coma Scale score in the RRC was 4. Although he did not undergo intubation and targeted temperature management (TTM), he had no neurological sequelae upon discharge. To the best of our knowledge, this report is the first to describe the case of a child with a decreased level of consciousness after near-hanging, who was treated without intubation and TTM.

## Introduction

Hanging is a common method of committing suicide in many countries, including Japan, South Korea, and Australia [1]. Hanging self-harm, described as "near-hanging" when the patient survives to arrive in the emergency department (ED), is a relatively rare trauma mechanism. In recent years, two large observational studies have failed to demonstrate an association between targeted temperature management (TTM) and survival or favorable neurological outcome, in patients after near-hanging [1,2]. While prehospital medical interventions are evolving and improving survival rates, the evidence for adequate early prognostic assessment is often insufficient. If the consciousness disorder is improving, it is difficult to decide whether we should intubate.

## Case presentation

A healthy, 12-year-old Japanese child was admitted to our hospital after near-hanging with reduced consciousness. His mother found him hanging from a ceiling. He had been hanging for up to 10 minutes with his legs fully suspended. His mother immediately untied him and laid him on the floor. The patient was pale, weak, and could not breathe, suggesting a cardiac arrest, and his mother initiated cardiopulmonary resuscitation (CPR) immediately. It took less than one minute from his mother's discovery to his rescue, and his mother's CPR confirmed the return of spontaneous circulation (ROSC) in three minutes. Three minutes after his mother found him, his sister called an ambulance, and the patient started breathing; thus, CPR was discontinued. When the ambulance arrived, he was breathing spontaneously, but his eyes were closed and he did not move. His heart rate was 106 bpm, blood pressure was 139/83 mmHg, respiratory rate was 20 and irregular, pupils were 4.0 mm bilaterally, and there was no light reflex. The electrocardiogram showed sinus rhythm. There was a rope scar on his neck, but no petechiae on the conjunctiva. Seven minutes after the ambulance arrived, the RRC also joined the ambulance. The patient's Glasgow Coma Scale (GCS) score was 4 (E1V1M2). Vital signs were unchanged, but pupils were 4.0 mm bilaterally and bilateral light reflexes had recovered. Since he was in a decerebrate posture, we judged it to be a seizure and administered midazolam. Subsequently, his muscle tone improved and seizures were ruled out, but his GCS score remained unchanged at E1V1M2. The patient transiently experienced shallow and slow breathing with a respiratory rate of about 15, temporarily requiring bag-valve-mask ventilation; however, the symptoms soon improved. A cervical collar was applied to prevent cervical spine injuries. His consciousness level improved (GCS score of 6 (E1V1M4)) on admission to the ED. Assuming the time at which his mother found him to be zero, it took 11 minutes for the ambulance to arrive, 18 minutes for the RRC contact, and 29 minutes for admission to the ED.

**Figure 1:**
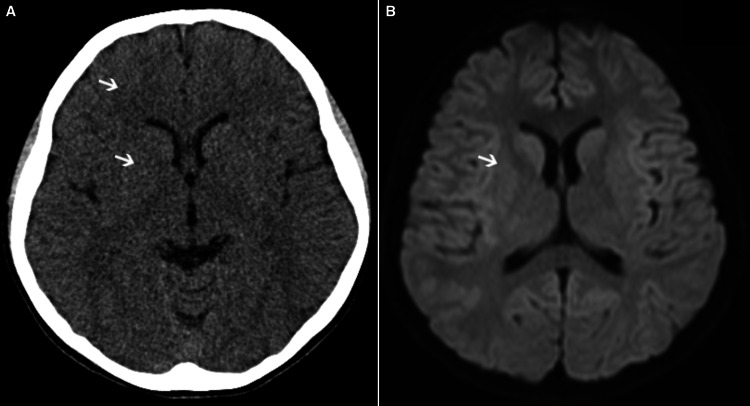
Representative image of the patient's brain (A) An initial plain CT scan. (B) Diffusion-weighted magnetic resonance imaging performed on Day 3. Both imaging showed no abnormalities. The arrows indicate that the normal structure of the brain is maintained.

Venous blood gas analysis at the ED showed a blood pH of 7.20, a partial pressure of carbon dioxide (pCO_2_) of 49.8 mmHg, and a lactate level of 7.2 mmol/L. Intubation was not performed because breathing was normal, i.e., respiratory rate of approximately 20, normal breathing pattern, no dyspnea, and no need for oxygen. The patient was admitted to the pediatric intensive care unit (PICU). Computed tomography (CT) on PICU admission showed no damage to the skull, cervical spine, and upper airway, and no pulmonary edema (Figure 1A). Normalization of arterial blood gas (pH: 7.32) was noted 115 minutes after his mother found him. However, after six hours, the level of consciousness remained low (GCS score of 7 (E1V2M4)).

Since he became restless and had increased movements of standing up and twisting his body, dexmedetomidine was started for the purpose of sedation. There were no blood sugar or electrolyte abnormalities at that time. And acetaminophen was regularly administered every six hours to avoid hyperthermia. On awakening the next morning (14 h after the patient was found), the GCS score was full and consciousness was clear. There were no abnormal findings on head magnetic resonance imaging (MRI) performed on Day 3, to confirm the absence of microscopic neurological damage (Figure 1B). However, because of acute mental illness, the patient was transferred to a psychiatric ward on Day 5, and referred to psychiatric treatment on Day 13. One month after onset, he had no neurological sequelae.

## Discussion

We are now able to perform intubation and TTM in critically ill patients without major adverse events. Doctors should avoid putting the patient's life at risk by not performing these treatments. However, in mild cases where we see clinical improvement in a few hours, we observe the patient closely and wait for the patient to recover, instead of treating them with intubation and TTM. Our report here describes this difficult decision based on our findings so far.

The intubation of patients with impaired consciousness is not mandatory. The American College of Surgeons Committee on Trauma Advanced Trauma Life Support recommends intubation for airway protection when the GCS score is ≤8 [3]. However, there is no direct evidence supporting this recommendation. In addition, intubation may be harmful in some cases, even when the GCS score is ≤8 [4]. Intubation is performed to prevent respiratory failure, provide respiratory support, and prevent the progression of brain damage. A study reported that 16% of pediatric patients did not require advanced airway management in the prehospital setting [5]. Given the risk of cervical spine and airway injuries, endotracheal intubation in pediatric cases involving near-hanging requires the support of an experienced anesthesiologist or otolaryngologist [6]. Therefore, care should be taken when intubating near-hanging victims.

It is necessary to assess the number and severity of near-hanging injuries. The frequency of disturbances of consciousness (GCS score of 8), pulmonary edema, and seizures in near-hanging victims is 54.5%, 28.5%, and 9%, respectively [7]. Cervical spine and upper respiratory tract injuries are also common in these patients [6]. In our case, acute seizures were treated with midazolam. There was no evidence of pulmonary edema on physical examination or CT imaging at presentation. Physical examination revealed no tracheal stenosis or cervical spine injuries affecting respiratory muscles.

In judging the necessity of intubation in the acute phase of this case, it is important to consider whether breathing is secured. Regardless of the GCS score, if airway, breathing, and circulation remain compromised, prompt intubation is required. This is because hypoxia and hypercapnia exacerbate brain damage. Respiratory depression occurred after midazolam administration and was resolved with temporary manual ventilation. In this case, no intubation was required.

In near-hanging cases, compression of the soft tissues of the neck leads to jugular vein occlusion, cerebral edema and ischemia, and unconsciousness. Loss of muscle tone leads to further constriction, carotid artery obstruction, cerebral hypoxia, airway obstruction, and death.

Targeted temperature management after ROSC suppresses necrosis and apoptosis during ischemia and reperfusion [8]. Several observational studies have shown that TTM for near-hanging does not improve mortality and neurologic outcomes [1,2].

Kim et al. reported that TTM might do harm to the patient after prehospital ROSC [1]. In the cases of ROSC with lay rescuers, it is not known whether the patient actually had a cardiac arrest. Loss of consciousness after near-hanging may be due to syncope from vagal stimulation or seizures from cerebral ischemia [7]. There is little evidence to support the use of TTM in pediatric patients after near-hanging because only one case report evaluated the effectiveness of this intervention in this population [8]. In our patient, a favorable neurological outcome was obtained without TTM.

The ineffectiveness of TTM may be related to the polarization of prognosis (either those who die or those with good neurological outcomes) depending on the initial state of the patient [9]. A single-center study identified the clinical factors associated with improved neurological outcomes in children after near-hanging and showed that patients with poor outcomes had lower blood pH (6.9 vs. 7.3) and lower GCS scores (3T vs. 14) [10]. All patients with worse neurological outcomes had a GCS score of 3T. In our case, the blood pH was 7.2 and the initial GCS score was 4. Moreover, our patient had a good neurological outcome, despite the slow improvement in consciousness. Thus, higher levels of consciousness may be associated with better neurological outcomes.

It is necessary to manage the airway, breathing, and circulation to ensure patient safety. Our patient did not require intubation, demonstrating that the intubation of pediatric patients with impaired consciousness after near-hanging can be avoided if the following conditions are met: stable spontaneous breathing, absence of upper respiratory stenosis, absence of pulmonary disorders such as pulmonary edema or pneumonia, absence of paralysis of the respiratory muscles, and a tendency toward improved consciousness. Additionally, it is also necessary to treat the patient in a safe place. The patient must be transported to a pediatric tertiary-care center, carefully monitored, and placed in a position to intervene promptly.

## Conclusions

A pediatric patient after near-hanging with an initial GCS score of 4 was treated without intubation or TTM and had no neurological sequelae after one month. Children who suffer near-hanging might be able to avoid intubation with careful observation if consciousness is improving and there are no respiratory or circulatory problems. However, further research is needed to identify the reasons why most other cases require invasive interventions.
